# PLAYgrounds: Effect of a PE playground program in primary schools on PA levels during recess in 6 to 12 year old children. Design of a prospective controlled trial

**DOI:** 10.1186/1471-2458-11-282

**Published:** 2011-05-08

**Authors:** Mirka Janssen, Huub M Toussaint, Mechelen Van Willem, Evert ALM Verhagen

**Affiliations:** 1 Academy of Physical Education, Technical University of Applied Sciences of Amsterdam, The Netherlands; 2 EMGO+ Institute for Health and Care Research, Department of Public & Occupational Health, VU University Medical Center, Amsterdam, The Netherlands

## Abstract

**Background:**

The relative number of children meeting the minimal required dose of daily physical activity remains execrably low. It has been estimated that in 2015 one out of five children will be overweight. Therefore, low levels of physical activity during early childhood may compromise the current and future health and well-being of the population, and promoting physical activity in younger children is a major public health priority. This study is to gain insight into effects of a Physical Education based playground program on the PA levels during recess in primary school children aged 6-12.

**Methods/design:**

The effectiveness of the intervention program will be evaluated using a prospective controlled trial design in which schools will be matched, with a follow-up of one school year. The research population will consist of 6-12 year old primary school children. The intervention program will be aimed at improving physical activity levels and will consist of a multi-component alteration of the schools' playground. In addition, playground usage will be increased through altered time management of recess times, as well as a modification of the Physical Education content.

**Discussion:**

The effects of the intervention on physical activity levels during recess (primary outcome measure), overall daily physical activity and changes in physical fitness (secondary outcome measures) will be assessed. Results of this study could possibly lead to changes in the current playground system of primary schools and provide structured health promotion for future public health.

**Trial registration:**

Netherlands Trial Register (NTR): NTR2386

## Background

The health benefits of an active lifestyle are well established [1-5]. Sedentary lifestyle habits are a major international and Dutch Public Health (PH) problem [1]. A recent study in the Netherlands revealed that less than 10% of the children in primary schools (ages 4 through 11 years) achieve 30 minutes of physical activity (PA) per day [6], while the guidelines state a minimum of 60 minutes of PA per day for children in this age range [7]. Although between the ages 12 and 17 this percentage triples to around 30%, the number of children meeting the minimal required dose of daily PA remains execrably low. In addition, children's PA has been identified as a modifiable risk factor for lifestyle related diseases such as coronary heart disease [7,8] and osteoporosis [7,9]. Therefore, low levels of PA during early childhood will compromise the current and future health and well-being of the population [10], and promoting PA in younger children is a major PH priority [1,6].

Schools have been recognized as key settings in promoting PA [6,10-13]. Next to the home, the school is the environment where children spent most of their time [6,11-13]. Within the school, physical education (PE) lessons and recess (i.e. regular playtime breaks) represent the two main contexts in which children have the opportunity to be physically active [6,11-13]. Next to such structured and frequent PA opportunities, schools can cater irregularly for sporting days and other extracurricular activities (e.g. swimming). For the promotion of PA in the school setting, interventions targeted at recess have an important advantage over other interventions. While other physical activities provided by the school are on an irregular or non-daily basis, during recess all children have the opportunity to be physically active every single day. In addition, it has been suggested that younger children are more likely to participate in moderate to vigorous PA within unstructured play settings as opposed to more structured contexts [14].

To stimulate PA in children different playgrounds in Dutch neighborhoods have been developed (e.g. Cruyff Court, Krajicek Playground, Nike Zone Parc). At these playgrounds children have an energy expenditure of about 206 kcal/hour and participate in moderate to vigorous PA 35% of the play time [15]. Unpublished pilot research for this study on other, not specifically stimulating, playgrounds show that children participate in moderate to vigorous PA 10-30% of the play time. One of the stimulating playgrounds, Nike Zone Parc, is placed on the school grounds to stimulate PA in children, but this playground costs about Euro 50.000.

Research in the United Kingdom in children aged 4-11 years, showed that the application of simple multicolored markings on the school's playground significantly increased children's participation in moderate and vigorous physical activities on both the short-term [12] and long-term [13]. In addition it was found that children who were less active at baseline benefited more from this intervention than their more active peers.

Such a simple and cheap intervention has great potential also in the Dutch setting. A recent Dutch study revealed that in primary school-aged children, PA levels are below recommended guidelines [6]. This especially holds true for neighborhoods in which the part of the population consisting of immigrants is relatively high [16]. The lower PA levels are suggested to be due to the lower level of participation in organized sports and PA. The latter is a major issue for girls, while they may not participate in organized sports and PA due to their religious or cultural beliefs. The ability to give such groups of children the opportunity to be physically active on a daily basis during school recess has great potential PH gain. Next to descriptive information, this Dutch study [6] also described that children in primary schools prefer a re-structuring of school playgrounds in order to make better use of the playground. In addition, the PE teacher has the ability to support physical activity further by linking the playground to the PE curriculum [15].

In this study playground alterations in combination with a supporting PE program will be evaluated.

### Objective

The objective of this prospective controlled trial is to gain insight into effects of a PE based playground program on the amount and level of PA in primary school children aged 6-12.

This overall aim can be subdivided into five research questions that will be addressed in this study:

(1) What is the effect of the playground program on the amount and level of PA during recess in primary school children aged 6-12?

(2) What is the effect of the playground program on the amount and level of daily PA in primary school children aged 9-12?

(3) What is the effect of the playground program on physical fitness in primary school children aged 9-12?

(4) Are there differences in the effect of the playground program on PA levels for children of different ages?

(5) Are there differences in the effect of the playground program in PA levels for boys and girls?

## Methods/Design

The CONSORT statement was followed to describe the design of this study [17].

This statement provides a checklist intended to improve the quality of reporting randomized controlled trials.

### Hypothesis

It is hypothesized that recess PA levels will increase as a result of the intervention. In addition, an increase in the amount of daily PA is hypothesized. As a result of the positive effects mentioned above, overall fitness is expected to improve. It is hypothesized that boys have higher PA levels than girls [15] and that PA levels of younger children will be increased more as a result of the intervention than PA levels of older children [15].

### Study outline

The PLAYground study is a prospective controlled trial with a follow-up of one school year (corresponding to approximately 9 months) in a group of about 1,200 children from 8 primary schools. Intervention and control schools will be matched according to number of pupils, geographical location, playground size and usage of the playground before the intervention.

Baseline measurements for playground usage take place before the curricular year (June), baseline measurements for fitness and overall PA take place at the start of curricular year (September). Follow-up measurements for PA take place in January (mid-year) and in June (end of the curricular year). Follow-up measurements for fitness and overall PA will also take place in June.

The study is funded by the board of the schools, the Stichting Westelijke Tuinsteden (STWT) and by the Academy of Physical Education, Technical University of Applied Sciences of Amsterdam (ALO). The study design, procedures and informed consent procedure are approved by the Medical Ethics Committee (2010/222; NTR2386) of the VU University Medical Centre, the Netherlands.

The research population will consist of 6-12 year old primary school children. All children at the participating schools will participate in this study following passive informed consent as has been used in comparable studies [18,19]. Schools will inform all parents on the study goals and procedures. If a parent does not want their child(ren) to participate in the study, this can be indicated, after which the child will be excluded from the study population.

### Sample size

For the power analysis data from a previous descriptive study on PA during school recess in primary schools was used [20]. This study showed that at most 40% of the children participate in at least moderate PA during school recess. Results from a pilot study revealed that a doubling of the percentage of children participating in at least moderate PA is feasible. In order to establish such an effect with a power of 90% and an alpha of 0.05, a total sample of 64 children split across two groups is needed.

While schools will serve as intervention units ideally a cluster effect should be taken into account when establishing group size. Assuming an intra-cluster correlation coefficient of 10% a study sample consisting of 8 schools (4 intervention schools and 4 control schools) is required. Based upon a careful low-end estimate of approximately 150 participating children attending at these primary schools, this will result in a sample of about 1,200 children.

### Recruitment

The eligible research population will consist of 6-12 year old children (n = 1,200). The board of schools (STWT) and the management of the schools already gave their approval for this study. All of the participating primary schools in the urban area of Amsterdam are located in neighborhoods with a relatively large part of the population consisting of foreign descent immigrants.

The schools will be recruited through the STWT. All schools that are part of the STWT will be informed about the study. Interested schools may receive additional information, after which they are free to choose to participate. Intervention and control schools will be matched according to number of pupils, geographical location, playground size and usage of playground before intervention. Schools that participate as control school first, will be offered the same intervention after this study in the following curricular year, when proven effective.

For the determination of playground usage the playgrounds will be observed according to a validated standardised protocol (SOPLAY) [21], which consists of observations on the quantity of use of the playground in general, quantity of use of the playground by different groups of interest (e.g. age and gender), type of PA activities, intensity of PA, and aspects related to the physical environment (e.g. weather conditions, accessibility and teacher presence). With the SOPLAY protocol the playground will be scanned for child density and playground use every five minutes. One scan, including notation, takes one minute. The total observation takes one hour, which consists of 12 scans. To generate a reliable result for every playground 5 observations per playground will be done in different weather conditions.

### Intervention

The intervention consists of a multi-component alteration of the schools' playground. The playground will be actually modified. In addition, playground usage will be increased through an altered time management of recess times, as well as a modification of the content of PE lessons. This intervention has been evaluated in a pilot study in 1 primary school from the same geographical area as the study setting.

Playgrounds of the intervention schools will be painted during the summer holidays according to the school's preference. An analysis of the existing playground will provide information for the new designs that will be applied to the playground. Examples of designs are a soccer field, a basketball set-shot area, a circle for circular activities, a dance area, a throw and catch area, a rope skipping area and a bounce area. By using a set of predefined markings a playground is re-created that is appealing to children of all age groups represented in this study.

The basic idea behind all modifications is to give structure to the playground. This will ensure that the available space is divided between children, such that compatible activities are clustered in the available space. In addition this benefits the choice for different games, as the setting of the games can be situated on the most ideal playground spot.

Some designs will be named as "hotspots". A hotspot is a place where the majority of children would like to play (e.g. a soccer field). Usage of hotspots will be spread over the different classes, so all children will have the opportunity to play at a hotspot once a week. Also the recess available time will be divided between the different classes. This will create more relative playground space and allows for more intensive play opportunity per child. Once a week teachers will be playing together with the children. Once a month parents will be invited to join the children at the playground.

A specific PE program will further support the intervention. Themes of activities will be scheduled and the regular lessons of PE will present ideas on how to use the playground, fitting the theme of the month (for example rope skipping in April). The weekly frequency of regular PE is two times with a duration of forty-five minutes per PE lesson. To further support the playground activities each class will have a box with playground attributes (for example ropes and balls) used in the physical activities.

### Outcome measures

The primary outcome measure of this study is PA levels of children during recess. Secondary outcome measures are overall daily PA and physical fitness.

Process outcome measures are: (1) factors that determine the success or failure of implementation of playground markings in primary schools; (2) enabling factors for a nation-wide implementation of playground markings in primary schools; and (3) possible explanations of the outcomes of the effect-evaluation. These process measures will be assessed by questionnaires, in personal interviews (with children, teachers, and parents) and observations.

### Measurements and follow-up

In order to register PA intensity during recess, each school will be visited every fortnight. Measurements will take place during this visit and will consist of both objective as well as observational measurements.

#### Accelerometers

PA will be measured using accelerometers (ActiGraph ActiTrainer), which are a reliable and valid objective PA measurement tool for children and adolescents [22]. During the researchers' fortnight visit of the schools, a total of 40 children of all ages (8 per grade) will be randomly chosen to wear an accelerometer during the school day. This is an arbitrarily chosen number, based upon the organisational ability to perform these measurements on a single day by the available research staff. The Actitrainer will be set at an epoch of 1 second to measure every change in intensity and the display will be turned off, so children will not be distracted by the Actitrainer. The Actitrainer will be called a 'growing meter' or a 'honesty meter', so children will not be stimulated to be more active just because of wearing the Actitrainer.

Through these measurements it is possible to objectively register intensity and duration of PA during recess. Existing analytical CSA programs (e.g. MAHUffe) are not usable to analyse the thus obtained data, as recess time only lasts 15 minutes. These current commercial programs are set to analyse PA during a prolonged period of time, e.g. 5 consecutive days. For this reason a dedicated Matlab program to analyse the number of counts during the short recess time was written. This Matlab program analyses the counts per minute for every child, and combines the resulting data with date of measurement, total time of measurement and grade, age and sex of the child.

### Questionnaires

A baseline questionnaire will be completed by the 9 to 12 year old children before they will perform the physical fitness test. This questionnaire gathers information about demographics (grade, age, gender), current PA levels (sports and leisure time). A follow-up questionnaire after nine months measures any changes in the baseline PA behaviour.

### Physical Fitness

Physical characteristics, i.e. body height, body weight as well as physical fitness, will be measured through a combination of items from the EUROFIT test [23] and the MOPER test [24]. The selected tests for this study are: one hand plate tapping, sit-and-reach, 10 × 5 Shuttle Run (MOPER and EUROFIT), standing broad jump, handgrip test, 20 m endurance Shuttle Run, anthropometry (body height, body weight) (EUROFIT) and bent arm hang. The bent arm hang is performed in the MOPER test and in the EUROFIT test with an elevated horizontal bar, but will be performed in this study at a rope instead of an elevated horizontal bar. Children step from a chair to bent arm hang in the rope and the time in bent arm hang will be recorded until the arms are not bent anymore. These tests give an overall image of the fitness of children (coordination, flexibility, endurance, strength and speed). The choice for these tests is based upon the organisational ability to perform these measurements within two PE lessons and to get a reliable result with different test leaders. A 20 m endurance Shuttle Run (EUROFIT) is performed instead of a 6 minute run (MOPER), because a warming-up is integrated in the Shuttle Run test. The flamingo balance test, the sit-ups in 30 seconds test (EUROFIT) and the leg lifting test (MOPER) have been left out because of the difficulty to get an objective result with different test leaders; the PWC170 (EUROFIT) has been left out, because a bike ergo meter test is not practical within two PE lessons; the vertical jump and the one-arm pull (MOPER) have been left out, because these tests measure the same construct as the standing broad jump and the handgrip test. The EUROFIT test and MOPER test have been shown reliable and valid in Dutch children [25] and have reference values for 9-12 (MOPER) and 12-16 year (EUROFIT) old children. Therefore in this study only 9-12 year old children will perform the Physical fitness test. The follow-up measurement after nine months measures any changes in the baseline physical fitness.

### Observations

During the fortnight's school visit, two researchers will observe the school's playground during recess. Observations will take place according to a validated standardised protocol (SOPLAY) [21], which consists of observations on the quantity of use of the playground in general, quantity of use of the playground by different groups of interest (e.g. age and gender), type of PA activities, intensity of PA, and aspects related to the physical environment (e.g. weather conditions, accessibility and teacher presence).

### Process evaluation

The PLAYground intervention will be evaluated with the use of the RE-AIM framework. The RE-AIM acronym represents Reach, Efficacy/Effectiveness, Adoptation, Implementation and Maintenance [26]. All children and teachers from the intervention group will complete an extra questionnaire and interviews on the subjective response of the intervention program and suggested improvements at follow up (January 2010 and July 2010).

### Statistical analyses

The effectiveness of the PLAYground intervention will be analysed by means of a multilevel regression analysis with the outcome measures at follow-up (9 months) as the dependent variables and adjusting for the baseline levels of the outcome measure. Both crude and adjusted analyses will be performed. Regression analyses will be performed using SPSS 18.0 (SPSS Inc. Chicago, Illinois, USA). For all analyses a two-tailed significance level of < 0.05 will be considered statistically significant.

## Discussion

The effects of the intervention on PA levels during recess (primary outcome measure) and overall daily PA and physical fitness (secondary outcome measures) will be assessed. Results of this study could possibly lead to changes in the current playground system of primary schools and may provide structured health promotion to enhance future public health.

## Competing interests

The authors declare that they have no competing interests.

## Authors' contributions

EALMV conceived of the idea for playground alterations. HMT and MJ obtained funding for the study. EALMV, HT and MJ developed the intervention, developed the design of this trial, and MJ recruited participants. WvM provided advice on the study design and contributed to the content of the article. MJ is the study investigator, was responsible for data acquisition, and wrote the article. All authors read and approved the final manuscript.

## Pre-publication history

The pre-publication history for this paper can be accessed here:

http://www.biomedcentral.com/1471-2458/11/282/prepub
